# Long-term data of the new transcutaneous partially implantable bone conduction hearing system Osia®

**DOI:** 10.1007/s00405-021-07167-9

**Published:** 2021-11-18

**Authors:** Ann-Kathrin Rauch, Thomas Wesarg, Antje Aschendorff, Iva Speck, Susan Arndt

**Affiliations:** Department of Oto-Rhino-Laryngology, Medical Center, University of Freiburg, Killianstr. 5, 79106 Freiburg, Germany

**Keywords:** Conductive and mixed hearing loss, Single-sided deafness, Active partially implantable transcutaneous bone conduction implant, Bone-anchored hearing system

## Abstract

**Purpose:**

The new active transcutaneous partially implantable osseointegrated system Cochlear™ Osia® System is indicated in case of conductive or mixed hearing loss (CHL/MHL) with a maximum average bone conduction hearing loss of 55 dB, or in single-sided deafness (SSD). The implant directly stimulates the bone via a piezoelectric transducer and is directed by an external sound processor. We conducted a monocentric retrospective longitudinal within-subject clinical study at our tertiary academic referral center. The aim was to investigate long-term data (2017–2021) on audiological outcomes and hearing-related quality of life for the Osia system.

**Methods:**

Between 2017 and 2020, 22 adults (18: CHL/MHL; 3: SSD) were implanted with the Osia100 implant; seven received bilateral implants. As of 10/2020, the sound processor was upgraded to Osia 2.

**Results:**

Mean Osia system use by 04/2021 was 30.9 ± 8.6 months (range 17–40 months). Unaided bone conduction thresholds were unchanged postoperatively. One patient had to be explanted because of prolonged wound infection. Aided hearing thresholds were significantly lower compared to the unaided thresholds preoperatively, along with a marked increase in speech recognition in quiet. Speech processor upgrade resulted in a stable benefit. Patients with CHL/MHL and SSD showed a similar improvement in self-rated hearing performance revealed by SSQ, APHAB, and HUI questionnaires.

**Conclusion:**

The Osia system is a safe, effective and sustainable option for treatment of conductive and mixed hearing loss or single-sided deafness.

## Introduction and background

The new active transcutaneous partially implantable bone conduction hearing system Osia® by Cochlear™ (Osia; Cochlear, Sydney, Australia) is indicated for conductive or mixed hearing loss (CHL/MHL). A maximum average hearing loss of up to 55 dB in bone conduction (BC) at the frequencies 0.5, 1, 2, and 4 kHz (4PTA_BC_) can be treated. This represents a large increase in maximum 4PTA_BC_ tolerable for a BAHS, especially when comparing to the first BAHSs, which were designed for a maximum 4PTA _BC_ of 35–40 dB [1]. Additionally, the Osia system is indicated in single-sided deafness (SSD) similarly to other BAHS [2]. The advantage of Osia with a larger gain at higher frequencies compared to passive BAHS has been reported [3, 4]. Therefore, we want to examine the functional and effective gain of Osia treatment in both CHL/MHL and SSD patients.

**Fig. 1:**
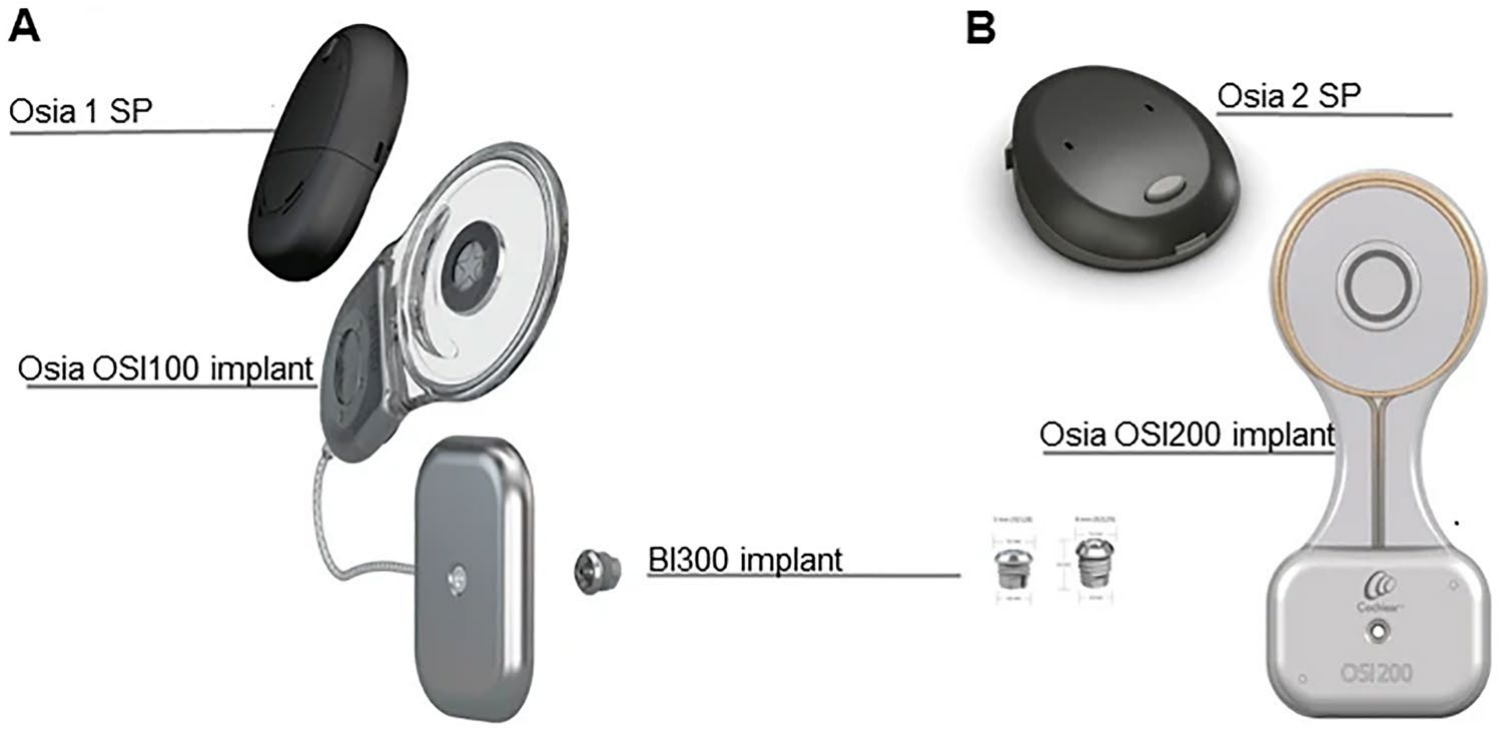
Cochlear™ Osia® System implants OSI100 and OSI200 with Osia 1 (**A**) and Osia 2 (**B**) SP. Illustration provided by Cochlear, Sydney, Australia

The osseointegrated implant directly stimulates the bone with its piecoelectric transducer and is directed by an external sound processor (SP). In October 2020, the new Osia system Osia 2 comprising the OSI200 implant and the Osia 2 SP was introduced by Cochlear. Since 10/2020, the SP was upgraded from Osia 1 to Osia 2 SP in Freiburg (Fig. 1). The first implantation of the new implant OSI200 in Europe was done in 04/2021 [5]. The Osia 2 has a reduced size and several additional features, such as automated setting of microphones, app directability, wireless compatibility and increased battery time [6]. Therefore, for the first time, this study compares outcomes after Osia SP upgrade in a large patient cohort.

Safety and effectiveness of the Osia system have been proved, e.g., by Lau et al. [7], and results from 12-month follow-up of a large cohort were published by Mylanus et al. [8]. However, long-term data above 12 months’ time have not been published to date, and we want to close this gap with our study assessing both objective and subjective outcomes of Osia implantation.

The aim of this study is to evaluate long-term outcomes beyond the first year of Osia: Do patients show lasting benefits in audiological outcomes, and is their hearing-related quality of life (QoL) improved? How is the functional and effective gainacross the frequency range of pure-tone audiometry? Does the SP upgrade from Osia 1 to Osia 2 result in a benefit for the patients? In addition, the following questions were addressed: Does the type of hearing loss (CHL/MHL, SSD) result in outcome differences? Is there a different benefit for uni- vs. bilateral Osia implantation? Are there factors that may predict postoperative speech recognition and self-reported QoL?

## Patients

### Patient collective and ethical statement

Between 2017 and 2020, 22 adult patients were implanted with the Osia system (OSI100 implant, Osia 1 SP; Fig. 1A). Ten of the patients (12 ears) participated in the CBAS5539 multicenter study [8]. Since the beginning of Cochlear’s controlled market release of the Osia system in 12/2019, we have implanted additional ten patients until 03/2021.

Our study was approved by the local ethics committee (No. 21-1142) and done in agreement with the Declaration of Helsinki (2013 version). Informed consent was obtained by all participating students prior to this study. This study was registered with DRKS (www.drks.de; No. DRKS00024640).

Visits were scheduled preoperatively and postoperatively at six and 12 months, and before and after the upgrade to the Osia 2 SP (Fig. 1B). By 04/2021, 19 out of the 22 patients had received an Osia 2 and nearly all (19/22) came to a follow-up visit at > 12 months of Osia use (Table 1). Most of the patients with previuos ear surgeries already had a CT or DVT scan preoperatively, verifying bone thickness at the implantation site to be at least 3–4 mm.

**Table 1:** Patient characteristics

ID	Sex	Age (yrs)	Etiology	Type of hearing loss	Side	Follow-up (mths)	LT
1	M	37	Recurrent ear surgeries for cholesteatoma, chronic otitis media	CHL	BIL	40	Yes
2	M	33	Chronic otitis media	SSD	R	40	Yes
3	M	27	Microtia °III	CHL	R	40	Yes
4	F	61	Tympanosclerosis	CHL	R	40	Yes
5	M	18	Aural atresia (Nager Syndrome)	CHL	BIL	40	Yes
6	M	41	Radical cavity, post cholesteatoma	CHL	L	2	No
7	M	52	Recurrent ear surgeries, post cholesteatoma	CHL	R	37	Yes
8	F	77	Recurrent ear surgeries, radical cavity, post cholesteatoma, blunting of ear canal	CHL	R	36	Yes
9	F	30	Recurrent ear surgeries, radical cavity, post cholesteatoma	CHL	R	36	Yes
10	M	39	Recurrent ear surgeries, post cholesteatoma, post soundbridge-implantation	CHL	L	36	Yes
11	M	58	Recurrent ear surgeries, bilateral chronic otitis media, unilateral radical cavity	CHL	BIL	29	Yes
12	F	64	Recurrent ear surgeries, chronic otitis media	CHL	BIL	28	Yes
13	M	40	Recurrent ear surgeries, radical cavity, post cholesteatoma, post BAHA	CHL	BIL	30	Yes
14	F	43	Otosclerosis	CHL	L	24	Yes
15	F	43	SSD	SSD	R	29	Yes
16	M	48	SSD	SSD	R	29	Yes
17	F	16	Cholesteatoma	CHL	L	6	No
18	F	69	Chronic otits media, down syndrome	CHL	L	18	Yes
19	F	57	Cholesteatoma	CHL	BIL	17	Yes
20	M	59	Chronic tube ventilation disorder	CHL	BIL	7	No
21	M	52	Jugular glomus tumor, obliteration of external auditory canal	CHL	L	18	Yes
22	M	11	Cholesteatoma, Cloves syndrome	CHL	L	17	Yes

*M* male, *F* female, *yrs* years, *CHL(MHL)* conductive/mixed hearing loss, *SSD* single-sided deafness, *side* refers to implanted side(s): *R* right, *L* left, *BIL* bilateral, *mths* months, *LT* long-term (included for study analysis of long-term use > 12 months)

## Methods

### Hearing thresholds

Pre- and postoperative unaided and aided bone conduction (BC) and air conduction (AC) thresholds at frequencies 0.25, 0.5, 0.75, 1, 2, 3, 4, 6, and 8 kHz were measured with headphones, and the contralateral ear was masked with narrowband noise. In all subjects, four-pure-tone average hearing threshold (4PTA) of both ears was determined for BC pre- and postoperatively, and for AC preoperatively. Additionally, aided thresholds were obtained using warble tones for BAHS (Baha BP110 or Ponto Pro Power) on a softband preoperatively (hereafter referred to by “BAHS condition”), and for Osia 1/2 postoperatively.

### Speech recognition in quiet

Speech recognition in quiet was assessed using the Freiburg monosyllabic test (“FR MS”) in the free field at 65 dB SPL preoperatively and postoperatively for all treated ears at 12 months’ time, before, and after SP upgrade, in best-aided condition each. Preoperative best-aided condition was defined as BAHS on softband (definition: “BAHS condition”). Masking of the contralateral ear was done using 70 dB broadband noise.

### Hearing-related QoL

The following questionnaires were completed by the patients preoperatively and postoperatively at 12 months, 24 months, and 36 months and used in statistiscal analysis if entirely completed: Speech, Spatial and Quality of Hearing Scale (SSQ), Health Utility Index (HUI) Mark2/3, and Abbreviated Profile of Hearing Aid Benefit (APHAB).

### Statistical analysis

Statistics were done using SPSS Version 27 (IBM Corp.). Analysis of differences in mean values for two groups was done with t-tests and Levene test for equality of variance; analysis of different factors was done using univariate ANOVA with Tukey post hoc tests. Level of significance was defined as < 0.05 (< 0.05: *, < 0.01: **, < 0.001: ***). Predictors were analyzed through linear regression analysis using ANOVA.

## Results

Data from 22 patients were available. 18 patients presented with CHL/MHL and 3 with SSD. Of the 19 patients, seven were bilaterally implanted. 19 out of the 22 patients had a follow-up visit at > 12 months postoperatively (26 ears) and were included into long-term follow-up analysis.

### Etiology

With respect to etiology (*n* = 22) of hearing loss, 13 patients had chronic otitis media, 3 SSD, and 3 ossicular dys-/aplasia, respectively. Otosclerosis (2) and tumor (1) were less frequent.

### Surgery and postoperative complications

Mean surgery time was 64.4 ± 23 min for unilateral Osia implantation, and 160 ± 49 min for bilateral implantation. There were two serious adverse events: One patient had to be explanted due to prolonged wound infection postoperatively (Patient No. 6 in Table 1). Another patient needed reimplantation because of wound infection at primary diagnosis of acne inversa, his postoperative audiometry results remained constantly good thereafter.

### Hearing thresholds (Fig. 2)

**Fig. 2:**
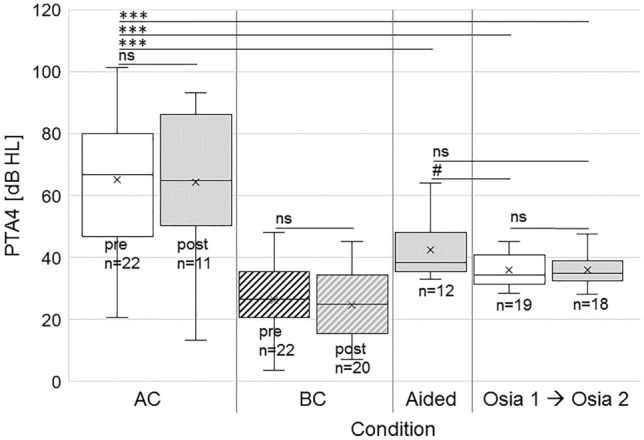
Box-whisker plots of 4PTA of CHL/MHL patients: Air conduction (AC) and bone conduction (BC) thresholds of the treatment ears remained stable after Osia implantation (> 12 months). Results in the BAHS condition and with Osia 1 and 2 SP postoperative showed significant benefit against the preoperative unaided situation. 4PTA with Osia 1 vs. 2 were not significantly different. Box-whisker plots are given for AC and BC pre- and postoperative, for BAHS condition, Osia 1 and 2 SP.Significance defined: *: ANOVA; #: Student’s *t* test

In CHL/MHL patients, 4PTA_AC_ (Fig. 2) were unchanged postoperatively with stable AC (*n* = 22/11 pre/post) and BC (*n* = 22/20 pre/post) thresholds (*p* > 0.05). One-way ANOVA and post hoc tests revealed a significant improvement in 4PTA_AC_ compared to preoperative (unaided) 4PTA_AC_ (mean 4PTA_AC_ unaided (*n* = 22): 65.3 ± 23.2 dB HL; mean 4PTA_AC_ BAHS condition (*n* = 11): 42.98 ± 9.95, Osia 1 (*n* = 19): 35.34 ± 5.11; Osia 2 (*n* = 18): 36.04 ± 5.22; *p* < 0.001 for all comparisons). The 4PTA_AC_ of the CHL/MHL patients obtained with Osia 1 and Osia 2 were not significantly different from BAHS condition preoperatively (*p* > 0.05, Fig. 2). In addition, paired t-tests showed improved 4PTA_AC_ with Osia 1 (*p* = 0.034) compared to BAHS condition preoperatively, while Osia 1 and 2 were not significantly different (*p* = 0.0683). 4PTA_AC_ did not differ significantly between Osia 2 and preoperative BAHS condition (*p* = 0.051). In summary, Osia implantation resulted in an improved 4PTA hearing threshold compared to the preoperative unaided and BAHS condition.

4PTA_AC_ and 4PTA_BC_ of treated ears in SSD patients (not shown) were unchanged postoperatively (mean value of unaided (*n* = 3): 123.33 ± 11.55 dB HL; BAHS condition (*n* = 1): 50; Osia 1 (*n* = 1): 41.5; Osia 2 (*n* = 3): 28.61 ± 19.95; all: *p* > 0.05). One-way ANOVA showed a significant effect of treatment condition on 4PTA. Due to the small number of the cohort, no post hoc analysis was performed. Student ‘s t-tests showed significant benefit from unaided compared to BAHS condition condition (*p* = 0.032), and compared to Osia 1 (*p* = 0.026) and Osia 2 (*p* = 0.005). 4PTA of BAHS condition vs. Osia 1 and Osia 2 was not significantly different. In summary, Osia implantation in SSD resulted in a significantly lower 4PTA.

### High vs. low frequencies (Fig. 3)

**Fig. 3:**
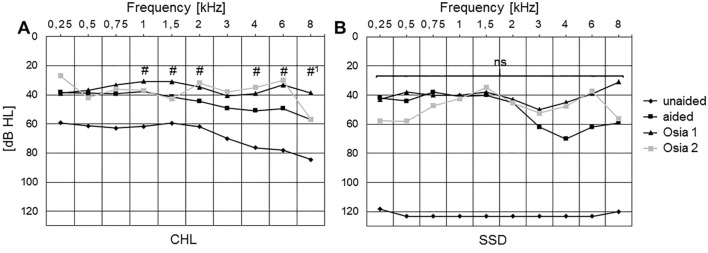
Mean air conduction hearing thresholds of CHL/MHL (A) and SSD (B) treatment ears for conditions unaided, BAHS condition, and with Osia 1/2: Osia 1 and Osia 2 SP showed a tendency for decreased (improved) thresholds compared to the preoperative unaided situation in high vs. low frequencies for both CHL (A) and SSD (B) patients. N: unaided CHL: 23, SSD: 3; BAHS condition CHL 11, SSD 1; Osia 1 CHL: 19, SSD: 1; Osia 2: CHL: 18, SSD:3. Levels of significance were determined for Osia 1/2 vs. BAHS condition. #: Student’s *t* test, #1: result applies to Osia 1 vs. BAHS condition only

Hearing threshold improved across low to high frequencies by Osia implantation compared to BAHS condition, with the largest benefit at higher frequencies (Fig. 3A: MHL/CHL; Fig. 3B: SSD). Across all frequencies, One-way ANOVA showed a benefit for CHL/MHL (Fig. 3A) in all test conditions (BAHS condition /Osia 1/Osia 2; at least *p* < 0.001 for all results) against the preoperative unaided situation. In CHL/MHL, there was a tendency for increased improvement at higher vs. lower frequencies for Osia 1/Osia 2 compared to the preoperative BAHS condition (*p* > 0.05; Fig. 3A); Osia 1 and 2 were not different from another (*p* > 0.05). *T* tests showed benefit for Osia 1 and Osia 2 vs. BAHS condition in nearly all frequencies above 1 kHz (1–8 kHz, except for 3 kHz, and for 8 kHz for Osia 2; all: *p* < 0.05). This was underlined by patient’s reports of increased benefit at higher frequencies by Osia implantation compared to the BAHS condition.

In SSD (Fig. 3B), across all frequencies, patients improved against unaided (BAHS condition /Osia 1/Osia 2), with a tendency for a greater benefit at higher frequencies (not significant; Fig. 3B).

### Speech recognition scores in quiet (Fig. 4)

**Fig. 4:**
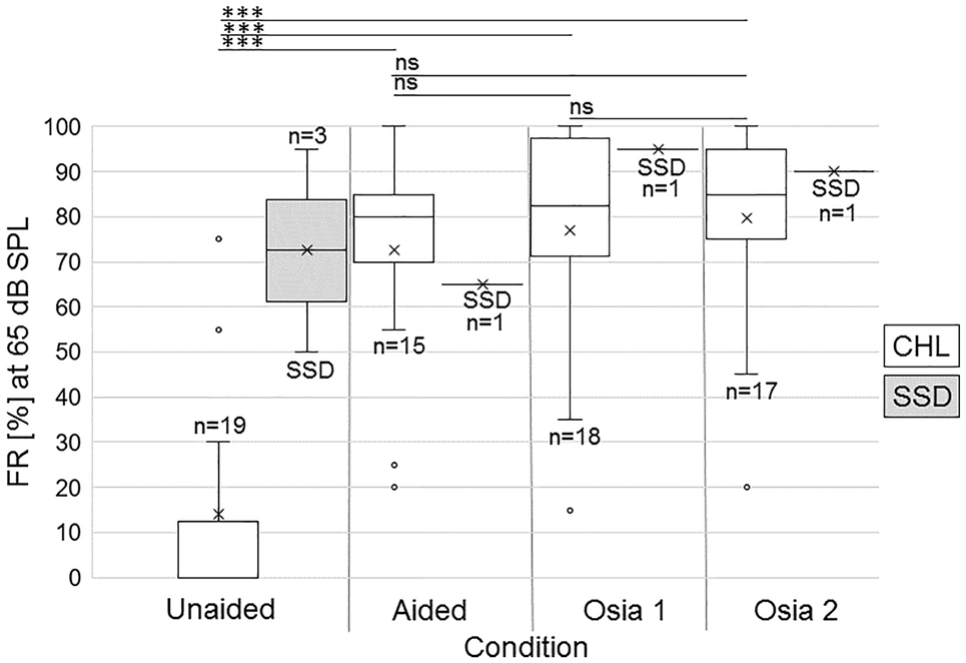
Box-whisker plots of speech recognition in quiet scores for all treated ears in the conditions unaided, BAHS condition, Osia 1/2: Results in the BAHS condition and with Osia 1 and Osia 2 SP showed significant benefit against the unaided situation. Results with Osia 1 vs. Osia 2 were not significantly different. Results are depicted for CHL and SSD patients separately (legend on the right side)

Compared to the preoperative unaided condition, monosyllabic speech recognition scores in quiet were significantly improved in the preoperative BAHS condition (13.9 ± 25.7%, *n* = 19) and with Osia 1/Osia 2 SP (mean of unaided (*n* = 19): 13.94 ± 25.74; BAHS condition (*n* = 15): 72.67 ± 22.9; Osia 1 (*n* = 18): 76.94 ± 23.9; Osia 2 (*n* = 17): 79.71 ± 20.8; all comparisons: *p* < 0.001; Fig. 3). Although average speech recognition scores were better for Osia 1 and Osia 2 compared to preoperative BAHS condition, and slightly better for Osia 2 vs. Osia 1 SP, results were not significant (*p* > 0.05).

In SSD patients, absolute speech recognition scores with Osia were improved compared to BAHS condition (unaided (*n* = 3): 72.5 ± 31.82; BAHS condition (*n* = 1): 65; Osia 1 (*n* = 1): 95; Osia 2 (*n* = 1): 90; results not shown; due to single results no statistical analysis performed).

To sum up, results of pure-tone and speech audiometry displayed a functional gain through Osia implantation. Osia 1/2 scores were better compared to BAHS condition (BAHA BP110/Ponto Pro on a softband).

### Hearing-related QoL (Fig. 5)

**Fig. 5:**
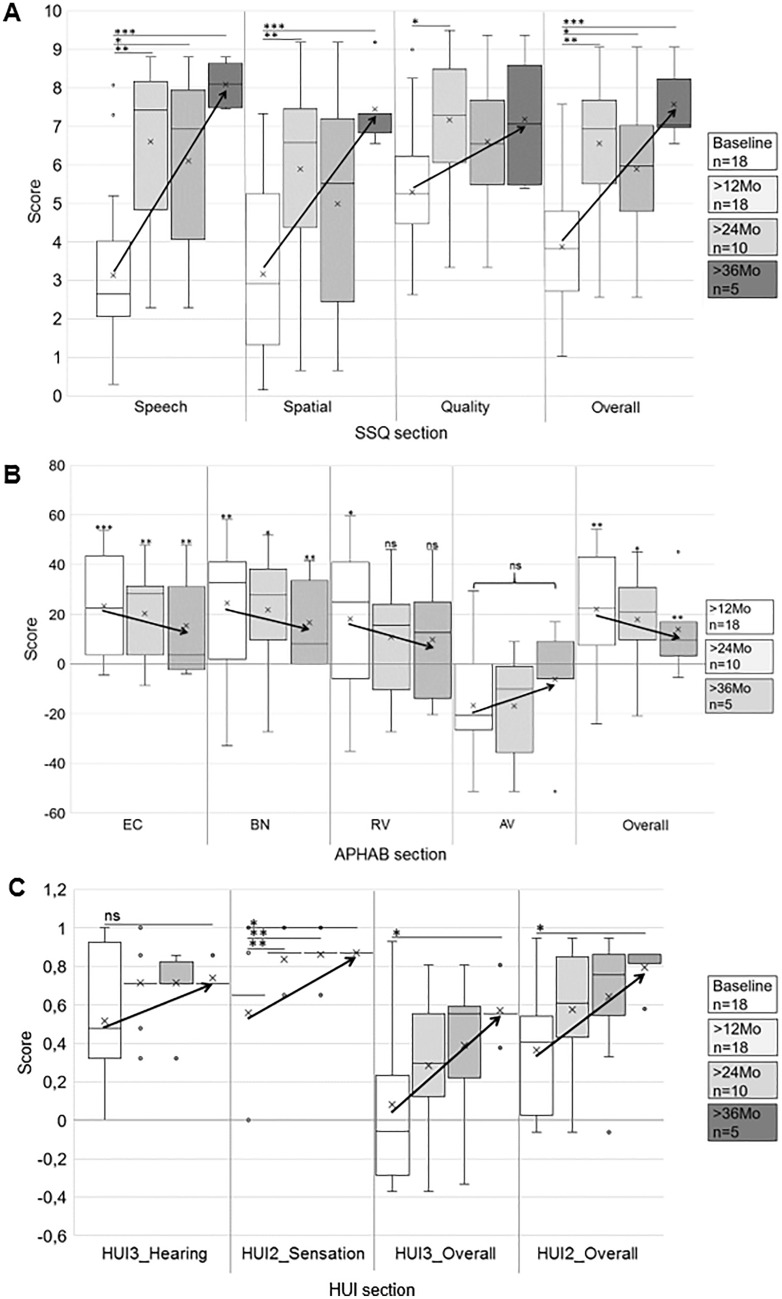
Box-whisker plots of subcategory and total scores of questionnaires SSQ (5A), APHAB (5B), and HUI (5C) with obtained preoperatively and with Osia at 12, 24, and 36 months (“Mo”, legend on right side). Arrows indicate the improvement in the mean value. A SSQ: patients showed benefit after Osia implantation in all sections, and the highest benefit at 36 months of Osia experience for speech, spatial, and overall score. B APHAB: subjects improved in sections ease of communication (EC), background noise (BN), reverberation (RV), and in overall APHAB score, but not in aversiveness to sound (AV), after Osia implantation. Referring to mean values, subjects tended to show increased benefit with rising Osia experience. C HUI: Osia implantation led to improvement in HUI2_Sensation, HUI3_Overall and HUI2_Overall; benefit in overall score was present at > 36 months of Osia experience. In HUI3_Hearing the patients, with respect to mean value, tended to improve (not significant)

All patients showed a significant benefit from Osia treatment compared to their preoperative situation revealed by all questionnaires assessing their hearing-related QoL. In SSQ (Fig. 5A), both CHL/MHL and SSD patients showed significantly better scores in all three categories and in overall score (comparisons with baseline (preoperative) *speech* (3.14 ± 2.1): 12 months (“Mo”): 6.61 ± 2.03 (*p* < 0.001), 24Mo: 6.11 ± 2.35 (*p* = 0.003), 36Mo: 8.1 ± 0.6 (*p* < 0.001); *spatial* 3.16 ± 2.29: 12Mo: 5.91 ± 2.56 (*p* = 0.008), 24Mo: 4.99 ± 2.88 (*p* = 0.242), 36Mo: 7.45 ± 1.03 (*p* = 0.006); *quality* 5.3 ± 1.78: 12Mo 7.18 ± 1.65 (*p* = 0.012), 24Mo: 6.61 ± 1.79 (*p* = 0.242), 36Mo: 7.19 ± 1.79 (*p* = 0.153); *overall score* 3.87 ± 1.84: 12Mo: 6.56 ± 1.79 (*p* < 0.001), 24Mo: 5.9 ± 2.07 (*p* = 0.033), 36Mo: 7.58 ± 1.04 (*p* = 0.001)).

Judging their QoL in the APHAB (Fig. 5B), patients revealed a significant benefit in categories ease of communication, background noise, reverberation, as well as in the overall score, while no significant improvement was achieved in the category aversiveness to sound (comparisons with baseline (preoperative) *EC* (34.89 ± 22.36): 12Mo: 11.58 ± 7.71 (*p* < 0.001), 24Mo: 11.03 ± 6.64 (*p* = 0.001), 36Mo: 8.93 ± 4.55 (*p* = 0.005); *BN* 53.32 ± 24.87, 12Mo: 28.9 ± 18.4 (*p* = 0.005), 24Mo: 30.67 ± 19.43 (*p* = 0.036), 36Mo: 15.2 ± 5.9 (*p* = 0.003); *RV* 47.6 ± 22.17: 12Mo: 29.51 ± 18.51 (*p* = 0.037), 24Mo: 36.47 ± 18.23 (*p* = 0.474), 36Mo: 24.3 ± 12.04 (*p* = 0.097); *AV* 23.11 ± 16.78, 12Mo: 39.81 ± 25.65 (*p* = 0.136), 24Mo: 39.32 ± 27.35 (*p* = 0.281), 36Mo: 24.57 ± 19.12 (*p* = 0.999); *overall score* 45.27 ± 21.43, 12Mo: 23.33 ± 12.92 (*p* = 0.001), 24Mo: 26.06 ± 13.5 (*p* = 0.021), 36Mo: 16.14 ± 4.72 (*p* = 0.005)).

In HUI2/3 (Fig. 5C), hearing- and sensation-related categories was analyzed as well as overall HUI2/3 score for all patients: In all categories but HUI3_Hearing, a significant improvement compared to preoperative was reached (comparisons by section: *HUI3_Hearing* vs. baseline (0.52 ± 0.38): 12 Mo: 0.72 ± 0.14 (*p* = 0.98), 24Mo: 0.72 ± 0.16 (*p* = 0.205), 36Mo: 0.74 ± 0.07 (*p* = 0.314); *HUI2_Sensation* vs. baseline (0.56 ± 0.33): 12Mo: 0.84 ± 0.11 (*p* = 0.001), 24Mo: 0.86 ± 0.08 (*p* = 0.004), 36Mo: 0.87 ± 0 (0.026); *HUI2_overall* vs. baseline (0.37 ± 0.32): 12Mo 0.58 ± 0.30 (*p* = 0.163), 24Mo: 0.64 ± 0.31 (*p* = 0.1), 36Mo: 0.8 ± 0.12 (*p* = 0.031); *HUI3_overall* vs. baseline (0.08 ± 0.43): 12Mo 0.28 ± 0.33 (*p* = 0.351), 24Mo: 0.39 ± 0.35 (*p* = 0.149), 36Mo: 0.57 ± 0.15 (*p* = 0.049)). HUI2_Sensation significantly improved with Osia at all follow-up intervals, as did the overall HUI2 and HUI3 scores at 36 months, but not at 12 or 24 months, of Osia experience.

For analysis of time points since implantation, it has to be noted that groups were overlapping (i.e., subjects who had their device for over 36 months were also included in the 24- and 12-months sections). Baseline, or preoperative, results for questionnaires existed for 18 patients. At follow-up of > 12 months, 18 patients had fully completed their questionnaires. For follow-up time of > 24 months, data from 10 questionnaires were available, and at > 36 months data from five patients were available.

In summary, results of SSQ, APHAB, and HUI questionnaires showed the largest benefit for the patients with the longest Osia experience above three years ‘ time.

### SSD patients benefit similarly compared to CHL/MHL patients in speech recognition and subjective evaluation

SSD patients all wore their device full day (self-report; > 8 h/day) and showed comparable benefit to patients with CHL/MHL regarding results in the FR and in questionnaires (*p* > 0.05, results not shown). Average gain in overall SSQ score was better for CHL/MHL patients than SSD patients (CHL: 2.97 ± 1.58 vs. SSD: 1.38 ± 0.19; *p* = 0.19), but not significantly different, as was true for APHAB [CHL: 27.45 ± 15.7 vs. SSD: 7.08 ± 3.5 (*p* = 0.95)]. Only in the background noise section of the APHAB, CHL/MHL patients gained more than SSD patients (CHL 31.79 ± 17.89 vs. SSD 1.17 ± 1.65; *p* = 0.033). For HUI, in HUI3 overall score a significant gain for CHL/MHL vs. SSD patients was apparent (CHL: 0.24 ± 0.43 vs. SSD: -0.45 ± 0.44; *p* = 0.047). This could not be shown for subsections of HUI3_Hearing, HUI3_Speech, HUI3_Cognition, HUI2_Sensation, and HUI2_overall score.

### Uni- vs. bilateral Osia implantation in CHL/MHL does not lead to different outcomes in speech recognition scores and subjective evaluation

Both uni- and bilateral CHL patients (Table 1) wore their device full day (self-report; > 8 h). And both audiometric and patient-related outcomes of bilaterally implanted patients with CHL/MHL were not significantly different from unilaterally implanted patients. With respect to 4PTA and analysis of the entire frequency range (Figs. 2, 3), results from uni- vs. bilateral CHL/MHL patients were not significantly different from one another compared to unaided (BAHS condition/Osia 1/Osia 2; One-way ANOVA; *p* > 0.05 for all comparisons, results not shown). Subjective evaluation showed no different benefit in SSQ overall score or subsections (average gain in SSQ overall score: unilateral: 2.99 ± 1.52 vs. bilateral: 2.9 ± 1.9; *p* = 0.917), neither in APHAB overall or its subsections (average overall score: unilateral 26.05 ± 14.09 vs. bilateral 30.24 ± 20.02; *p* = 0.225) nor in HUI overall scores or subsections (average overall score, e.g., in HUI2: unilateral 0.24 ± 0.39 vs. bilateral 0.21 ± 0.15; *p* = 0.899).

### Sound processor upgrade from Osia 1 to Osia 2 SP

Results for average hearing thresholds (4PTA, Fig. 2), frequency-specific hearing thresholds (Fig. 3), and monosyllabic speech recognition scores (Fig. 4) were not affected by the sound processor upgrade from Osia 1 to Osia 2 SP (*p* > 0.05 for all comparisons). However, all patients preferred the new Osia 2 over the old Osia1 SP, subjectively regarded their hearing (“hearing quality” and “loudness”; self-reports) as better with the new SP, and had improved mean values in the aforementioned sections for Osia 2.

### Is the Osia experience a predictor for speech recognition scores and hearing-related QoL?

Increased time since implantation, or experience with Osia, could not predict increase in speech recognition (*p* > 0.05). Multiple regression analysis showed that age, but not Osia experience nor etiology of deafness, neither uni- vs bilateral Osia implantation, could predict monosyllabic speech recognition with Osia (*F* (2,16) = 4.51; *p* = 0.028; age (years): regression coefficient − 0.74, *p* = 0.013; all other factors: *p* > 0.05). For subjective evaluation (questionnaires), results were different: Increasing age predicted increased improvement in SSQ overall score compared to the preoperative situation (*F* (2,15) = 16,68; *p* < 0.001; age (years): regression coefficient 0.06, *p* < 0.001; Osia experience: *ns*) and in APHAB overall score (*F* (1,16) = 16,29; *p* = 0.001; age: regression coefficient: 0.37; *p* = 0.003), i.e., elder patients showed more benefit through Osia. Multiple regression analysis revealed no significant difference in HUI results with Osia compared to preoperatively unaided. With respect to type of hearing loss, comparing SSD vs. CHL/MHL patients did not result in differences predicting speech recognition scores and hearing-related QoL. Equally, subset analysis of uni- vs. bilateral implantation revealed no significant difference in predictors. Overall, Osia experience—independent of uni- or bilateral implantation, and independent of SSD or CHL patients—could not predict speech recognition benefits. Younger patients gained larger improvement of their hearing thresholds by Osia, while elder patients evaluated their subjective benefit through Osia implantation as higher compared to their younger counterparts.

## Discussion

SSD and uni-/bilateral CHL Osia recipients showed improved audiological and subjective outcomes after implantation throughout long-term follow-up of > 12 to > 36 months, which demonstrates the treatment to be safe, effective, and with lasting improved outcomes.

### Long-term data, surgery, and fitting procedure

These are the first long-term Osia data with a follow-up period of > 12 months. We can use this long-term data to better counsel patients with SSD or CHL/MHL (chronic otitis media with condition after several ear surgeries, ossicular dys-/aplasia, or in certain cases with otosclerosis) for treatment with an Osia. Surgery and fitting procedures are safe and reliable with a very low complication rate [8]. Crowder et al. (2021) reported number of soft tissue injuries to be lower with Osia compared to other BAHS as revealed by data from the Food and Drug’s Administration database, while complication rates as related to device failure were similar [9]. Our study confirms the complication-free surgery and low postoperative complication rate. We recommend an adapted surgical procedure in reducing the retroauricular bump in order to avoid discomfort and enhance the esthetic outcome [5]. The transcutaneous design with its reduced risk for skin infections – compared to the percutaneous approach of other BAHS – while providing similar audiometric benefit is very useful.

### Audiometric benefit, advantage at higher frequencies, and improvement in speech recognition with the Osia system comes along with improved subjective evaluation

The Osia system showed an increased 4PTA of around 7 dB against the preoperative BAHS condition (BAHS on a softband), along with significant improvement especially at higher frequencies. This matches the findings by Goldstein et al. (2020), in which Osia 2 resulted in an average additional 4PTA gain of around 10 dB to Baha Attract/Connect in > 40 surgeries [10]. Regarding the amplification at higher frequencies, which are decisive for speech perception, Goycoolea et al. (2020) found the Osia system to be superior to Baha®5 Super Power on a softband especially at 2–6 kHz [4]. We could confirm the large amplification power at higher frequencies and these findings, for the first time, for both CHL (uni- and bilateral) and SSD Osia recipients, who had a significant advantage in the frequency range of > 1 kHz by comparison with BAHS condition, i.e., BAHS on a softband. It should be mentioned that BAHS on a softband, because of cushioning through skin flaps, is inferior to in percutaneous BAHS.

Speech recognition scores in quiet were sustainably improved at > 12 to > 36 months of follow-up in our study. Osia does also provide a benefit for speech recognition in noise [4, 8]. Subjective evaluation using the SSQ, APHAB, and HUI showed benefit for Osia recipients [8, 11–13], and our long-term data revealed the largest benefit at > 36 months of Osia experience.

Gawecki et al. (2020) showed higher speech recognition scores as well as larger self-reported QoL with the Osia compared to the Baha Attract [11], while Pla-Gil et al. (2021) compared a control group of ten patients using a Baha 5 Power Connect with a group of 10 Osia 1 patients and found a comparable hearing performance in terms of both audiological and subjective outcomes [13]. As a consequence, Osia can be regarded as similarly effective treatment to other percutaneous BAHS with the highest audiological output, and as superior to other transcutaneous bone conduction devices (Ellsperman et al. (2021) provided an overview [14]).

### Benefit for both CHL/MHL and SSD patients and bilateral Osia

We have added evidence that both CHL/MHL and SSD Osia recipients ameliorate on both objective (audiological outcomes) and subjective level (patient-reported outcomes). Auditory rehabilitation of SSD patients with the Osia system is similarly possible as in CHL.

Binaural benefit has been shown for bilateral BAHS users in quiet and in noise [15–17], assumingly mainly through the head shadow effect, but also as improvement in real binaural hearing, i.e., sound localization [18]. A preliminary study in two of our bilateral Osia users revealed that they could make use of temporal gaps for speech recognition in noise (unpublished; Arndt and Wesarg (2019) Poster, International Congress on Bone Conduction Hearing and Related Technologies, Miami, FL). In our cohort, similar outcomes in audiological and subjective evaluation demonstrate that uni- and bilateral Osia implantation led to a similiar benefit. Further studies should exploit the extent of binaural benefits in larger groups of bilateral Osia recipients for speech performance in noise and sound localization.

### Sound processor upgrade to the Osia 2 SP

Upgrade to the Osia 2 SP was accompanied by a stable audiometric benefit compared to the Osia 1. Our results did not reveal an objective improvement through SP upgrade to the Osia 2—although the latter disposes of enhanced technical features. A benefit in speech recognition by SP upgrade had been shown, e.g., in CI patients [19]. However, in the present study, patients had a rather short experience with the Osia 2 SP at time of audiological examination compared to their previous Osia 1 SP, and might need more time getting used to the new SP. Still, all subjects reported advantages of the consistently preferred Osia 2 SP for speech recognition, esthetic outcome, and technical benefits.

### Are there factors predicting the outcome with Osia?

Füllgrabe et al. (2014) showed that younger age predicted enhanced speech recognition ability in a pair of IQ-, education-, and audiogram-matched groups of young and old normal hearing subjects, while self-rating of hearing performance did not change with age [20]. We demonstrate that in our group of Osia recipients, younger age also predicted better speech recognition score. Self-report of quality of hearing, however, was rated better with increasing age. In our view, changed cognitive abilities might explain the effect of age on perception of hearing ability.

### Strenghts, limitations, and outlook

Future studies should investigate long-term outcomes of Osia patients in terms of speech recognition in noise and localization abilities, especially in bilateral Osia implantation, and should take cognitive abilities, especially in elder candidates, into account. The Osia as a new active transcutaneous bone conduction device is recommendable for both patients with SSD and CHL. Similar to other BAHS, it is MRI conditional (surgical removal of magnet at 3.0 Tesla required) with creation of a local image artefact, which has to be taken into consideration when counseling patients and caregivers. Its large amplification power at high frequencies with enhanced speech recognition and stable subjective benefit at > three years of follow-up make it a safe and effective, transcutaeneous BAHS.

## Conclusion

In summary, the Osia system can be recommended as a safe and effective treatment for conductive and mixed hearing loss and in single-sided deafness. It combines the advantage of a transcutaneous solution, lowering the risk of recurrent wound infection, with a similar audiological gain compared to percutaneous BAHS. Bilateral Osia recipients showed comparable benefit to unilateral users. Upgrade to the newer generation of the Osia 2 SP introduced in 10/2020 led to a similar audiological benefit. Patients sustainably improved at > 3 years of Osia experience on both audiological and subjective evaluation.

## Data Availability

The data were pseudonymized for analysis. The study data are available from the corresponding author at the Department for Otorhinolaryngology, University Medical Center Freiburg, Germany and is saved in a separate study folder at the internal local computer network.
