# Correction: Long-term data of the new transcutaneous partially implantable bone conduction hearing system Osia^®^

**DOI:** 10.1007/s00405-024-08521-3

**Published:** 2024-03-26

**Authors:** Ann-Kathrin Rauch, Thomas Wesarg, Antje Aschendorff, Iva Speck, Susan Arndt

**Affiliations:** Department of Oto-Rhino-Laryngology, Medical Center, University of Freiburg, Killianstr. 5, 79106 Freiburg, Germany

**Correction: European Archives of Oto-Rhino-Laryngology (2022) 279:4279–4288** 10.1007/s00405-021-07167-9

In the original publication of the article, under the section “Hearing‑related QoL (Fig. 5)”, in the beginning of third paragraph, the following sentence “In all categories but HUI3_Hearing …” was published incorrectly. The correct sentence should read as follows: “Absolute scores improved in all categories. Significant improvemens were shown for HUI2_Sensation.”

Further, in the same paragraph, the phrase which denotes the values of “HUI2_overall vs. baseline” was published incorrectly as follows: “HUI2_overall vs. baseline (0.37 ± 0.32): 12Mo 0.58 ± 0.30 (p = 0.163), 24Mo: 0.64 ± 0.31 (p = 0.1), 36Mo: 0.8 ± 0.12 (p = 0.031); HUI3_overall vs. baseline (0.08 ± 0.43): 12Mo 0.28 ± 0.33 (p = 0.351), 24Mo: 0.39 ± 0.35 (p = 0.149), 36Mo: 0.57 ± 0.15 (p = 0.049)). HUI2_Sensation significantly improved with Osia at all follow-up intervals, as did the overall HUI2 and HUI3 scores at 36 months, but not at 12 or 24 months, of Osia experience.” The correct phrase should read as follows: “*HUI2_overall* vs. baseline (0.68 ± 0.18): 12Mo: 0.78 ± 0.2 (*p* = 0.38), 24Mo: 0.82 ± 0.2 (*p* = 0.241), 36Mo: 0.92 ± 0.05 (*p* = 0.07); *HUI3_overall* vs. baseline (0.51 ± 0.27): 12Mo: 0.59 ± 0.27 (*p* = 0.777), 24Mo: 0.65 ± 0.34 (*p* = 0.577), 36Mo: 0.83 ± 0.07 (*p* = 0.123)).”

Under the same section, on page 4285, the following sentence was published incorrectly as “In summary, results of SSQ, APHAB, and HUI questionnaires showed the largest benefit for the patients with the longest Osia experience above three years ‘ time.” The correct sentence should read as follows “In summary, results of the questionnaires, showed the largest benefit for the patients with the longest Osia experience above three years’ time.”

Similarly, under the section “SSD patients benefit similarly compared to CHL/MHL patients in speech recognition and subjective evaluation”, the following sentence “For HUI, in HUI3 overall score a significant gain for CHL/MHL vs. SSD patients was apparent (CHL: 0.24 ± 0.43 vs. SSD: -0.45 ± 0.44; p = 0.047). This could not be shown for subsections of HUI3_Hearing, HUI3_Speech, HUI3_Cognition, HUI2_Sensation, and HUI2_overall score.” was published incorrectly and should have been removed.

Likewise, under the section “Uni‑ vs. bilateral Osia implantation in CHL/MHL does not lead to different outcomes in speech recognition scores and subjective evaluation”, the following phrase “(average overall score, e.g., in HUI2: unilateral 0.24 ± 0.39 vs. bilateral 0.21 ± 0.15; p = 0.899).” was published incorrectly and should have been removed.

Finally, in Figure 5C, score values were published incorrectly. The correct Figure 5 and respective caption is provided in this article.

**Fig. 5:**
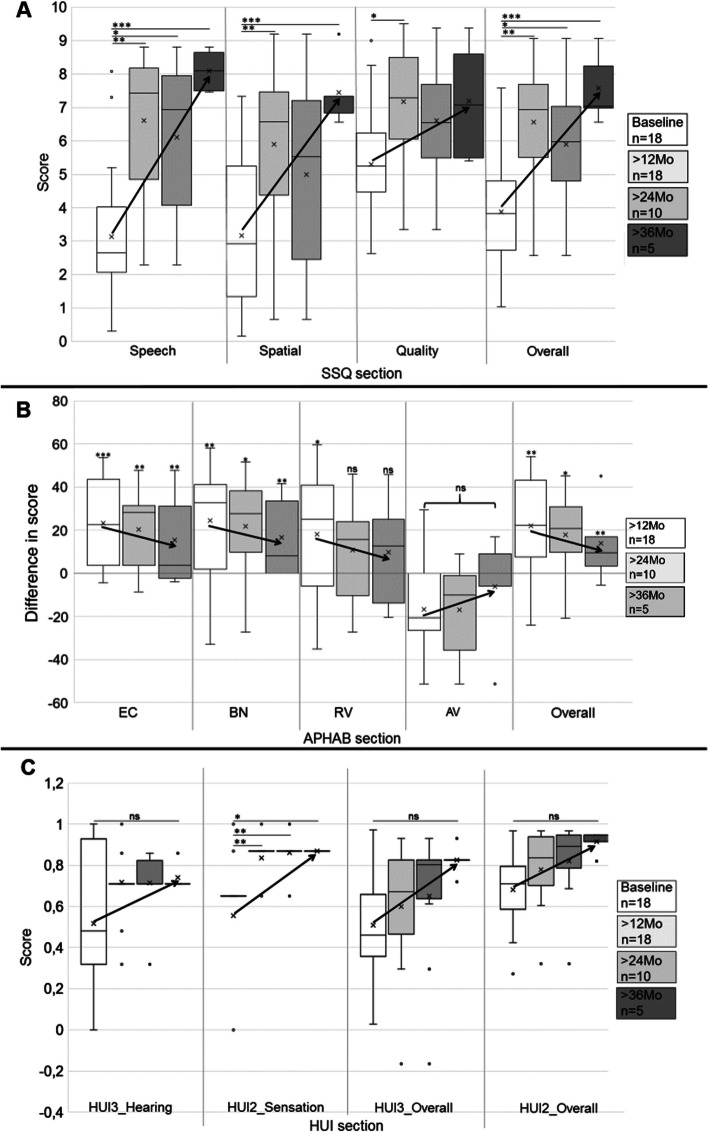
Box-whisker plots of subcategory and total scores of questionnaires SSQ (5**A**) and HUI (5**C**) obtained preoperatively and with Osia at 12, 24, and 36 months as well as differences in suibcategory and total APHAB scores (5**B**) between Osia at 12, 24, and 36 months and preoperatively unaided (“Mo”, legend on right side; "x": mean values). Arrows indicate the improvement in the mean value. A SSQ: patients showed a significant benefit after Osia implantation in all sections, and the highest benefit at 36 months of Osia experience for speech, spatial, and overall score. B APHAB: subjects improved significantly in sections ease of communication (EC), background noise (BN), reverberation (RV), and in overall APHAB score, but not in aversiveness to sound (AV), after Osia implantation. C HUI: Osia implantation led to a significant improvement in HUI2_Sensation. In HUI3_Hearing, HUI3_Overall and HUI2_Overall, the patients, with respect to mean value, tended to improve (not significant)

